# Identifying key genes for European canker resistance in apple: machine learning and gene expression profiling of quantitative disease resistance

**DOI:** 10.1038/s41598-025-33478-6

**Published:** 2025-12-27

**Authors:** Amanda Karlström, Antonio Gómez-Cortecero, John Connell, Charlotte Florence Nellist, Matthew Ordidge, Jim M. Dunwell, Richard Jonathan Harrison

**Affiliations:** 1 NIAB, Lawrence Weaver Rd, Cambridge, CB3 0LE UK; 2 School of Agriculture, Policy and Development, University of Reading, Reading, RG6 7EU UK; 3 Swedish University of Agricultural Sciences (SLU), Sundsvägen 14, Alnarp, 234 56 Sweden; 4 Wageningen University and Research , Wageningen, 6708 Netherlands PB; 5 School of Biosciences , University of Birmingham , Edgbaston, Birmingham , B15 2TT United Kingdom

**Keywords:** European canker, Apple, Neonectria ditissima, Transcriptome, *Malus* x *domestica*, Machine learning, Biotechnology, Computational biology and bioinformatics, Genetics, Molecular biology, Plant sciences

## Abstract

European canker, caused by *Neonectria ditissima*, is a major disease of apple (*Malus* × *domestica*) with limited control options, making host resistance a key management strategy. Although quantitative disease resistance (QDR) has been identified, the underlying molecular basis remains poorly understood. We investigated candidate genes associated with resistance using transcriptomic profiling of a bi-parental population segregating for six QTLs linked to canker resistance. RNA sequencing combined with machine learning enabled the identification of key biomarkers predictive of disease resistance. Integration of expression and QTL data highlighted genes involved in phenylpropanoid biosynthesis, immune receptors (NLRs, RLKs, WAKs), and epigenetic regulators, implicating their roles in host defense. Expression patterns were further resolved into cis- and trans-regulatory effects, providing insight into allele-dependent regulation. Independent validation in a separate dataset confirmed the robustness of key expression patterns. These findings advance understanding of the genetic architecture underlying QDR in apple and provide a basis for marker development to support breeding of cultivars with durable resistance to European canker.

**Supplementary Information:**

The online version contains supplementary material available at 10.1038/s41598-025-33478-6.

## Introduction

 European canker, caused by the ascomycete *Neonectria ditissima*, severely affects apple (*Malus* x *domestica*) production worldwide and is particularly problematic in cool, wet climates^1^. The fungus primarily colonizes woody tissues, entering via pruning cuts or other injuries on shoots and trunks, and then spreading internally through the vascular system^2^. *N. ditissima has* a wide host range and is able to infect a large number of decidious tree species^1^.

Preventive measures, including sanitation and fungicide applications, provide limited control of disease establishment, making the development of genetically resistant cultivars the most effective management strategy. Despite this, there is limited information on the response of the host apple plants to infection nor is there information on the resistance mechanisms involved in limiting the spread of the disease. Reported sources of partial resistance to *N. ditissima* in *Malus* are all of quantitative nature^3–7^. Several quantitative trait loci (QTL) with relatively small to moderate effects, in the range of 4–19%, have been reported to contribute to partial resistance to European canker in apple scion material. Together, these findings indicate that resistance to *N. ditissima* is polygenic and reflects the combined influence of multiple loci rather than the action of a single major gene. Interestingly, a number of quantitative disease resistance (QDR) alleles are also present in cultivars considered susceptible, such as ‘Gala’, which highlights that accumulating or “stacking” several favourable alleles may be required to achieve stronger levels of tolerance.

Despite its prevalence, the molecular basis of QDR remains poorly defined. In contrast, the functions of many resistance (*R)* genes that confer qualitative or race-specific resistance are well characterised^8^. Most of these have been shown to belong to the nucleotide-binding site leucine-rich repeat (NLR) family, which plays a central role in pathogen recognition^8^. Genes associated with QDR are functionally diverse and include those encoding kinases, WRKY transcription factors, zinc-finger proteins, and enzymes involved in lignin biosynthesis as well as NLRs^8^. Cell-surface receptors with extracellular sensing domains are also thought to play an important part in quantitative resistance, as they mediate the perception of pathogen-derived molecules at the plasma membrane^8^. These include receptor-like kinases (RLKs), receptor-like proteins (RLPs), LysM-domain receptors, lectin-type RLKs (LecRLKs), and wall-associated kinases or kinase-like proteins (WAKs/WAKLs)^9^.

A study investigating the host response of apple trees to *Valsa mali*, a necrotrophic fungal pathogen with a mode of infection and biology similar to *N. ditissima*, demonstrated that *V. mali* infection activates genes involved in plant-pathogen interactions, plant hormone signal transduction, flavonoid biosynthesis, and phenylpropanoid biosynthesis^10^. The phenylpropanoid pathway plays a crucial role in the synthesis of secondary metabolites, initiated by the deamination of phenylalanine to cinnamic acid via phenylalanine ammonia-lyase (PAL)^11^. Cinnamic acid serves as a precursor for the production of lignin, suberin, coumarins, flavonoids, and stilbenes, which contribute to plant defense mechanisms^12^. Similar host responses have been observed in poplar trees infected with canker pathogens^13,14^. We hypothesize that apple trees infected with European canker exhibit comparable responses. However, studies on the genetic responses of trees to fungal wood pathogens remain limited, despite their critical significance in forestry and horticulture.

Approaches to identifying genes underlying QDR include bulk transcriptome profiling to examine gene expression in resistant and susceptible hosts^15^ or contrasting individuals carrying resistant or susceptible alleles at a QTL^16–18^. Machine learning (ML) is emerging as a valuable tool to identify transcriptional predictors of plant immune responses^19^ and gene markers for classifying resistant and susceptible genotypes using transcriptome data^20^. Algorithms like Random Forest and Support Vector Machines identify key resistance-associated biomarkers by analysing gene expression patterns, while feature selection methods enhance model accuracy and biological interpretability.

We embarked on this transcriptome sequencing project not only to deepen our comprehension of the molecular mechanisms guiding the interaction between *N. ditissima* and apple but also to improve our understanding of the broader host responses to infection by fungal wood pathogens. Furthermore, our goal was to pinpoint candidate genes associated with QDR to facilitate the development of apple cultivars with heightened resistance.

We analyzed transcriptomic responses in a bi-parental offspring population segregating for six additive QTLs previously linked to disease resistance^5^. Individuals were grouped into ‘resistant’ and ‘susceptible’ bulks based on canker disease phenotypes, and gene expression patterns were identified using random forest-based variable selection and differential expression analysis.

We examined gene expression differences regulated by each QTL, focusing on both cis and trans variation. Our aim was to identify genes within QTL haplotypes with differential expression between resistant and susceptible alleles, either constitutively or in response to pathogen challenge. Gene expression patterns were validated in ‘Golden Delicious,’ which carries at least one copy of each QDR-associated haplotype and shares partial ancestry with the studied population through its offspring, ‘Gala.’, which was used as a parent in the segregating population^5^.

In this stepwise approach, we aim to understand both the general mechanisms of resistance and how specific elements of the host’s response to pathogen invasion are modulated in the presence of additive QTL.

## Methods

### Plant material

A subset of progeny from one of the families used for QTL identification by Karlström et al.^5^ were subject to transcriptome sequencing: 25 progenies from a cross between ‘EM-Selection 4’ x ‘Gala’ as well as the two parents were grafted in six replicates on ‘M9 EMLA’ rootstocks in January 2019 at NIAB, East Malling. All graftwood used was obtained from trees maintained at NIAB, East Malling. The trees were kept in pots in an unheated polytunnel and drip-irrigated for the full duration of the experiment.

Genotype bulks consisting of the ten most resistant (bulk-R) and ten most susceptible (bulk-S) progeny were selected based on recorded disease phenotype in the field experiment reported in Karlström et al.^5^.

‘Golden Delicious’ and ‘M9 EMLA’ trees used for validation and prediction were grafted onto ‘M9 EMLA’ rootstocks in January, 2017 at NIAB, East Malling, United Kingdom. The trees were maintained in pots in an unheated greenhouse and irrigated weekly. Six replicates were propagated. No trees showed symptoms of canker prior to the experiment. The trees in both experiments were kept in 2 l pots fertilised with a slow-release fertiliser (Osmocote).

### Artificial inoculation with *Neonectria ditissima* and sampling

The progeny trees were artificially infected in the unheated polytunnel in December, 11 months after grafting. The temperature and humidity in the polytunnel were not controlled or recorded”.

Four replicate trees of each genotype were inoculated with *N. ditissima* spore suspension and two trees mock-inoculated with a water control (Fig. S1).

Inoculation in the GD and M9 trees was carried out under different conditions compared to the progeny. At the end of July, six months after grafting, the ‘GD’ and ‘M9’ trees were moved to a chilled glasshouse four days prior to being inoculated. The glasshouse conditions were the following: temperature 15–25℃, relative humidity ≥ 80%. Misting lines were installed under the benches with trees on top in order to maintain the humidity. These were equipped with 360° misting units spraying water for one minute at ten minute intervals. Three replicate trees of each variety were inoculated with either a spore suspension or with a control consisting of water (Fig. S1).

Inoculations and the preparation of inoculum were performed as per Gomez-Cortecero et al.^3^. A single spore isolate of *N. ditissima*, Hg199, was used. Two leaf positions were inoculated (positioned at the 15 and 30th node from the apex) but only the top infection-point was used for sequencing. Each tree was inoculated by removing two leaves and the corresponding axillary bud with a scalpel and thereafter adding 3 µl of spore suspension with a concentration of 10^5^ macroconidia/ml to the wound with a pipette.

Progeny trees were sampled four months post-inoculation, when the majority of inoculated trees showed symptoms. Samples from lesions on progeny trees that had still not developed symptoms at 8 months post inoculation were removed. Samples from ‘GD’ and ‘M9’ were collected at 25 days post-inoculation, by which time symptoms had appeared for all inoculated trees^21^. In both experiments samples were taken shortly after the majority of trees showed the first emergence of canker symptoms. Stem samples were approximately 5 × 3 mm and included transverse tissue sections from the cortex, phloem, cambium and xylem of each tree. Samples from infected trees were collected at approximately 0.5 cm distance above the leading edge of the developing canker lesion. Mock-inoculated plant samples, hereafter referred to as ‘control’ samples, were taken from healthy wood, 0.5 cm from the point of water inoculation. All samples were taken apically in relation to the point of inoculation. Samples were flash frozen upon collection and stored at −80$^\circ C$ until RNA extraction.

### RNA-extraction and transcriptome sequencing

The frozen stem samples were ground using DEPC-treated pestle and mortars in the presence of liquid nitrogen. Total RNA was isolated using Qiagen RNeasy Plant Mini Kit (Qiagen Inc., Valencia, CA) according to instructions from the manufacturer.

One sample per tree was sequenced. Sequencing was performed by Novogene (Novogene, Hong Kong and Cambridge) on Illumina HiSeq 4000.

### Processing of sequence data and genome alignment

Adaptor sequences and low-quality data were removed from sequencing reads using fastqc-mcf^22^. RNA-seq data quality was evaluated using the quality control tool FastQC version 0.10.1^23^. Quantification of the expression of transcripts was done using Salmon version 0.9.1^24^ using the ‘GD’ transcriptome GDDH13 version 1.1.

### Analysis of differential expression in partially resistant and susceptible bulk

Differential expression (DE) analysis was performed in R (R version 4.5.1) using packages edgeR^29^ and limma (version 3.52.1)^30,31^. Initially, transcripts with low expression in the experimental samples were removed from the dataset. edgeR was used to calculate normalisation factors. Multidimensional scaling (MDS) plots were used to visually inspect the clustering of samples. Differential expression analysis was conducted by using function voom in package limma. Voom transforms raw counts to log_2_ counts per million reads (CPM), incorporating the normalisation factors. A correlation factor was added to the linear model fit in limma-voom to account for a higher degree of correlation between samples derived from the same genotype, thus enabling comparisons both within and between apple genotypes. Thresholds of log_2_ Ratio | ≥ 1 and a Benjamini-Hochberg (BH) adjusted *p*-value of ≤ 0.05 were used to determine if a gene was to be considered DE.

Five contrasts were used to identify differentially expressed genes (DEGs):

- Infected vs. Control for bulk-R genotypes.
- Infected vs. Control for bulk-S genotypes.
- Bulk-R vs. bulk-S for infected plants. This contrast was used to identify transcriptional differences between the groups during infection.
- Bulk-R vs. bulk-S for control plants. This contrast aimed to reveal constitutive transcriptional differences between the two groups under control conditions.

### Variable selection and classification using random forest

Random Forest (RF) classification implemented in R (packages randomForest and caret) was used to identify genes whose expression profiles best distinguished resistant and susceptible apple genotypes. Classification was performed on 72 inoculated samples, derived from 10 resistant and 10 susceptible genotypes within a full-sib progeny (33 resistant and 39 susceptible infected trees in total). The five progeny genotypes with intermediate resistance level were not used for this analysis. DEGs from any of the reported contrasts were included as predictors in the initial RF models.

To avoid confounding due to shared genetic background, a 5-fold cross-validation blocked by genotype was implemented, ensuring that all samples from a given genotype were assigned to the same fold. Within each training fold, 200 RF models were fitted using different random seeds to assess the stability of variable importance scores. Genes were ranked by their mean permutation importance across these repetitions, and the top 50 most informative genes were retained within each fold. Each model was then retrained using only the selected genes and evaluated on the held-out genotypes, yielding an unbiased estimate of predictive performance.

To evaluate feature stability, the frequency with which each gene appeared among the top 50 predictors across all folds was calculated. Genes selected in ≥ 80% of folds were considered stable predictors of resistance and were used for functional interpretation.

To further test the predictive capacity of the selected genes, model performance was evaluated using an independent RNA-seq dataset from three inoculated trees of the partially resistant cultivar ‘GD’ and three of the partially susceptible ‘M9 EMLA’^3,27,28^. Performance metrics were computed using the caret package, including accuracy, class sensitivity, specificity, and precision.

### Functional annotation and enrichment analysis

Predicted genes in the GDDH1.1 genome were annotated with Kyoto Encyclopedia of Genes and Genome (KEGG) and protein family (PFAM) terms. First, FASTA sequences for all genes were obtained from the Genome database for Rosaceae^32^. The gene annotation was thereafter performed in eggNOG-mapper 2.1.7^33^.

Gene set enrichment analysis was conducted in clusterProfiler^34^ for KEGG and PFAM terms. The list of background genes considered in the enrichment analyses was limited to genes that were expressed within the experiment. Terms with BH adjusted *p*-value of ≤ 0.05 were considered to be enriched. Only KEGG pathways that were represented in *M. x domestica* in the KEGG PATHWAY Database^35^ are presented.

### Comparative analysis of QTL-R and QTL-S transcriptomes

Progeny from the ‘EM Selection-4’ x ‘Gala’ cross were grouped based on the presence/absence of specific SNP-haplotypes at six genetic loci linked to QDR to European canker^5^. Individuals with the QDR haplotype are denoted QTL + while those lacking the haplotype are denoted QTL-. The DE analysis to identify candidate genes within QDR QTL was performed as described above.

1. The following contrasts were used to identify differentially expressed genes:
  - QTL- Control vs. QTL + Control: This contrast aimed to reveal constitutive transcriptional differences between the two groups under control conditions.
  - QTL- Infected vs. QTL + Infected: This contrast was used to identify transcriptional differences between the groups during infection.

The analysis did not differentiate between individuals with one or two copies of the QDR haplotype. The DEGs for each QTL with a genome position within the QTL interval were further explored. The physical position of the QTL regions were defined by the genome position of the boundary SNPs identified in Karlström et al.^5^. InterPro^36^ was used to provide further information on putative gene function for validated genes.

To determine whether there was a correlation between presence/absence of haplotypes at different QTL a chi-square test (chisq.test in base R) was performed for each pair-wise combination.

### Validation of gene expression in an independent experiment

Independent validation of gene expression was performed by contrasting control vs. infected trees from ‘GD’. DEGs from ‘GD’ was further used to validate candidate genes underlying QTL. ‘GD’ was used as a parent for QTL discovery by Karlström et al.^5^ and has at least one copy of each haplotype associated with the QDR QTL.

## Results

### Transcriptome profiling of apple trees upon *N. ditissima* infection using RNA-Seq

We sequenced the transcriptomes of a full-sibling progeny segregating for partial resistance QTL to *N. ditissima* during disease infection to investigate transcriptional responses linked to quantitative resistance. A mean library size of 18.5 Mb was obtained and 32,353 transcripts were retained after filtering out transcripts with low expression. A total of 146 samples were included in the final analysis after the removal of samples that showed unusual MDS plots compared to other replicates of the same genotype. For prediction and validation, the average number of reads mapped to the reference genome was 48 and 49 million for infected respectively uninfected ‘GD’ trees, and 46 and 51 million for infected ‘M9’ trees.

### Differential gene expression depending on resistance level to European canker

**Fig. 1:**
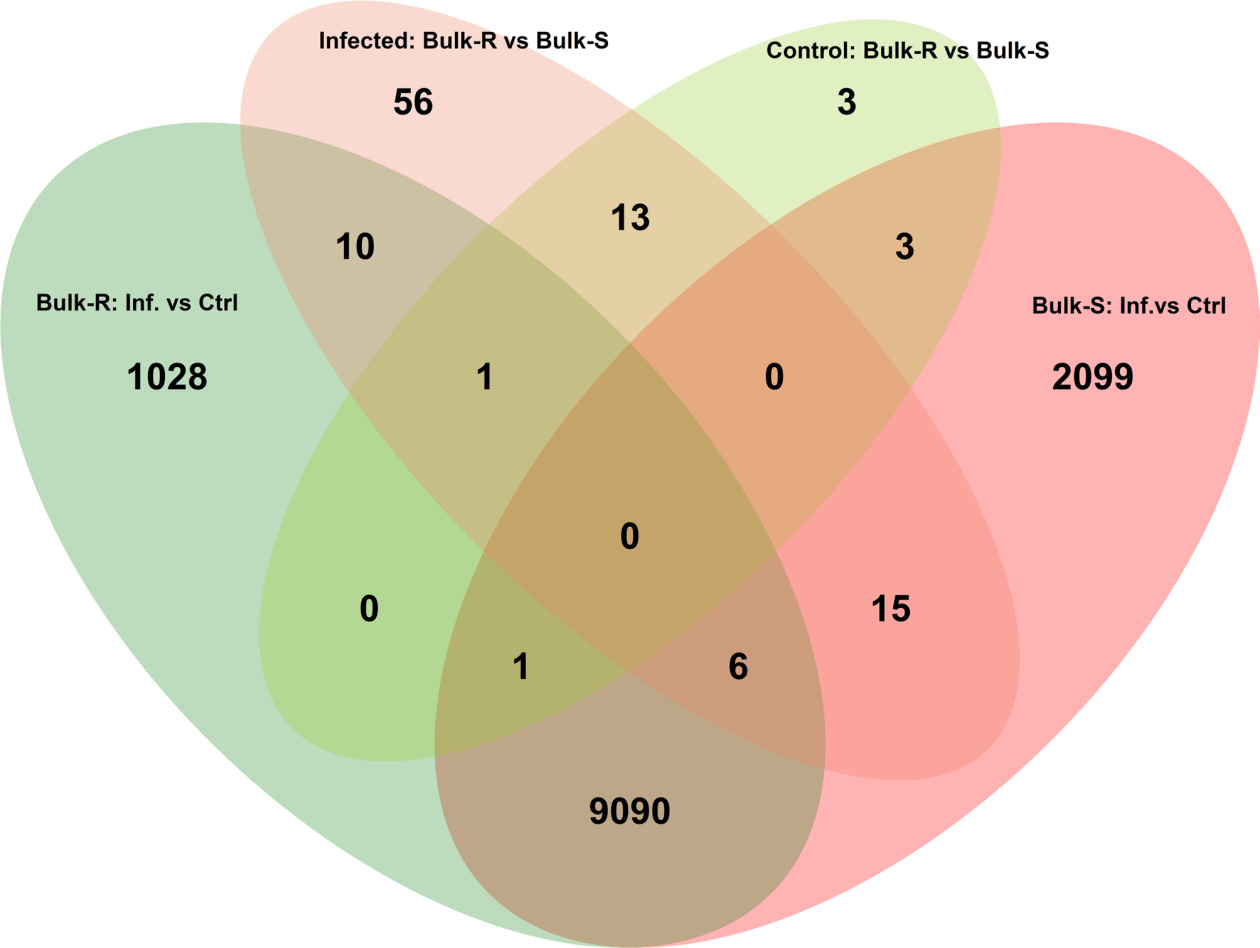
Venn diagram illustrating differentially expressed genes (DEGs) in 10 partially resistant (bulk-R) and 10 susceptible (bulk-S) apple genotypes from the same F1 progeny. The diagram shows DEGs derived from both the comparison between bulks (Bulk-S vs. Bulk-R) and within bulks (Infected vs. Control).

To investigate the differences in gene expression patterns between resistant and susceptible genotypes, a DE analysis was performed. This included within-group comparisons of infected versus mock-inoculated trees for ten partially resistant (bulk-R) and ten susceptible (bulk-S) genotypes, as well as between-group comparisons of bulk-R and bulk-S in both infected and control trees (Fig. 1, Table S1).

A total of 101 DEGs were identified from the comparison of infected bulk-R vs. bulk-S. Nine out of these transcripts were also DE in the partially resistant ‘GD’ upon infection (Table S1). Among the gene models identified in both experiments were a putative Lec-RLK (*MD05G1263100*), ABC-transporter protein (*MD03G1172200*) and a RLK (*MD00G1101400*).

The 30 DEGs with largest difference between bulk-R and bulk-S are shown in Table 1.

**Table 1:** The 30 DEGs with largest log2 fold-change between infected trees of bulk-R and bulk-S.

Gene ID GDDH13_v1.1	Chromosome	log2 fold-change (LFC)	BH adj. *p*-value	Predicted gene function	DE in ‘GD’ Infected vs. ‘GD’ Control
*MD10G1115400*	10	−4.97	0.009	TUNAMYCIN INDUCED 1-like	No
*MD10G1129900*	10	−4.36	0.001	Small conductance mechanosensitive ion channel	No
*MD10G1299100*	10	−3.85	0.019	Polyphenol oxidase	No
*MD01G1010300*	1	−3.81	0.011	SAM dependent methyltransferase	No
*MD02G1025000*	2	−3.79	< 0.001	Non-coding RNA*	No
*MD10G1137400*	10	−3.58	< 0.001	Disease resistance protein (NLR)	No
*MD03G1172200*	3	−3.33	0.001	ABC-2 type transporter	Yes
*MD10G1141100*	10	−3.12	0.001	Disease resistance protein (NLR)	No
*MD15G1267400*	15	−2.91	0.001	Translocase of outer membrane	No
*MD10G1248700*	10	−2.91	0.002	Coatomer	No
*MD10G1120500*	10	−2.82	< 0.001	Unknown function	No
*MD05G1213100*	5	−2.8	0.041	Ankyrin repeat family protein	No
*MD00G1101400*	NA	−2.79	0.021	Cysteine-rich receptor-like protein kinase	Yes
*MD15G1239400*	15	−2.69	< 0.001	LRR receptor-like serine threonine-protein kinase	No
*MD00G1169900*	NA	−2.66	0.019	Interferon-related developmental regulator (IFRD)	No
*MD05G1187500*	5	3.64	0.013	Non-coding RNA*	Yes
*MD10G1086300*	10	3.56	< 0.001	Unknown function	No
*MD05G1214700*	5	3.28	0.026	G-type lectin receptor-like kinase (LecRLK)	No
*MD10G1177500*	10	3.1	0.001	2-hydroxy-palmitic acid dioxygenase mpo1-like	Yes
*MD10G1306300*	10	3.06	0.002	AGAMOUS-like 24	No
*MD04G1002200*	4	3.03	< 0.001	Non-coding RNA*	No
*MD15G1252900*	15	2.95	0.001	Non-coding RNA*	No
*MD10G1122300*	10	2.88	0.001	Cyclase-associated protein	No
*MD10G1278800*	10	2.79	0.026	Myb family transcription factor PHR1-like	No
*MD03G1121500*	3	2.74	0.045	Phenylalanine ammonia-lyase (PAL)	No
*MD15G1294100*	15	2.67	0.049	Non-coding RNA*	No
*MD10G1283900*	10	2.6	0.001	HXXXD-type acyl-transferase	No
*MD10G1160000*	10	2.57	0.041	SUPPRESSOR OF AUXIN RESISTANCE 1-like	No
*MD05G1217400*	5	2.43	0.043	G-type lectin receptor-like kinase (LecRLK)	No
*MD01G1113000*	1	2.43	0.003	Magnesium transporter	No

Ten transcripts were identified at the intersection of the ‘Bulk-R Infected vs. Control’ and ‘Infected: Bulk-R vs. Bulk-S’ contrasts (Fig. 3, Table S2), indicating that they were induced upon infection and exhibited differential expression between resistant and susceptible genotypes. Of these, only one transcript, *MD05G1187500*, was DE upon infection in the partially resistant genotype ‘GD’. Although *MD05G1187500* lacks a predicted gene function, a BLAST search revealed strong sequence similarity to predicted non-coding RNA sequences in *Malus* sp. (E-value = 0.0, sequence similarity > 99%). Among the ten transcripts, two were putative NLRs (*MD04G1015300* and *MD10G1018400*), both of which showed lower transcript abundance in infected tissue and were less expressed in bulk-R genotypes. Additionally, a putative LecRLK, *MD10G1177500*, was DE in both comparisons, with increased expression in infected bulk-R compared to control bulk-R and higher abundance in bulk-R than in control (Table 1).

Thirteen predicted genes were identified at the intersection of the ‘Infected: Bulk-R vs. Bulk-S’ and ‘Control: Bulk-R vs Bulk-S’ contrasts, indicating they were constitutively different between bulk-R and bulk-S genotypes regardless of infection status (Fig. 1, Table S1). None of these transcripts were found among the DEGs in ‘GD’, which is consistent with the finding that they are constitutively expressed and not induced upon infection. Among the putative gene functions of these gene models were one NLR (*MD10G1137400*), two transcription factors (*MD10G1276200* and *MD10G1278800*), two with functions within RNA-processing (*MD10G1268700* and *MD10G1232900*) and four transcripts predicted to be non-coding RNA. Notably, ten of the 13 transcripts were located on chromosome 10.

### Variable selection in random forest for classification of resistant and susceptible Apple genotypes

Random Forest classification was applied to identify genes whose expression profiles best discriminated resistant and susceptible apple genotypes within a full-sib progeny. Using a genotype-blocked 5-fold cross-validation approach, in which all samples from a given genotype were assigned to the same fold, models trained on the top 50 most informative genes achieved an overall prediction accuracy of approximately 0.67 (95% CI = 0.55–0.77; *p* = 0.021, Table 2), significantly higher than expected by random classification (No Information Rate = 0.54).

**Table 2:** Predictive performance of random forest models using the top 50 most informative genes under genotype-blocked 5-fold cross-validation, and independent validation of the 26 stable genes in the partially resistant ‘GD’ and partially susceptible ‘M9 EMLA’ genotypes. *Asterisk indicates that model accuracy is significantly greater (*p* < 0.05) than the no information Rate, representing the accuracy expected by always predicting the majority class.

Performance metric	Independent validation (26 stable genes, ‘GD’ & ‘M9’)	Genotype-blocked 5-fold cross-validation (top-50 genes)
Accuracy	1.00 *	0.67 *
95% CI (Accuracy)	0.54–1.00	0.55–0.77
Sensitivity (Resistant)	1.00	0.64
Specificity (Susceptible)	1.00	0.69
Cohen’s Kappa	1.00	0.33

**Fig. 2:**
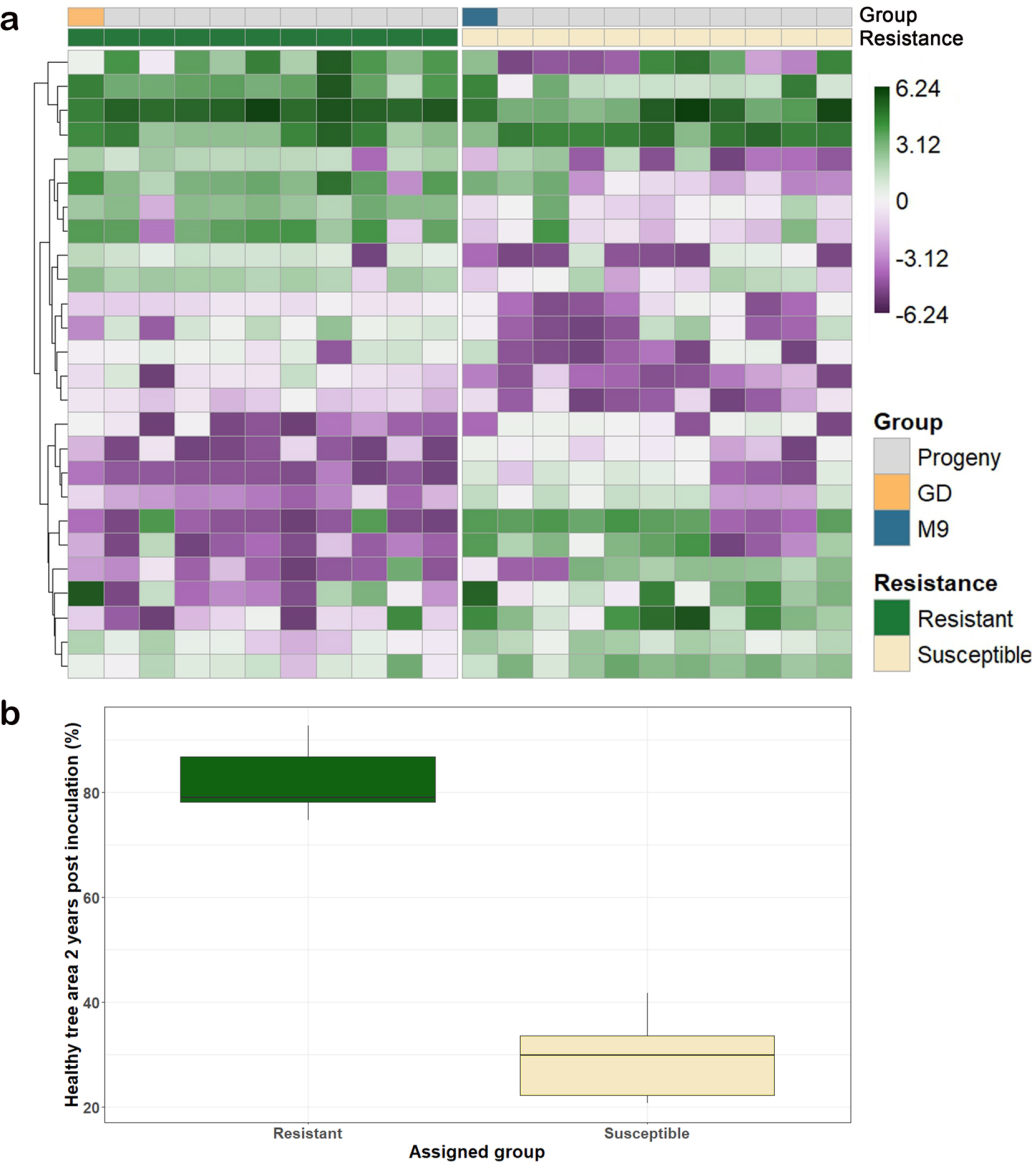
A Heatmap showing normalized read count data for the 26 transcripts selected as transcriptional predictors of canker resistance. Mean logCPM expression per genotype is presented for 20 full-sibling progeny, along with ‘Golden Delicious’ (GD) and ‘M9’. (B) Phenotypic classification of the 20 full-sibling progeny as ‘resistant’ or ‘susceptible,’ based on the characterization by Karlström et al^5^.

Across folds, 26 genes were consistently ranked among the top predictors in ≥ 80% of cross-validation folds, and were therefore considered stable transcriptional markers of resistance (Fig. 2; Table S3).

The predictive capacity of these 26 stable genes was further evaluated using an independent RNA-seq dataset from inoculated trees of the partially resistant cultivar ‘GD’ and the partially susceptible ‘M9 EMLA’ (Table 2).

### Functional categorisation of stable gene features

**Fig. 3:**
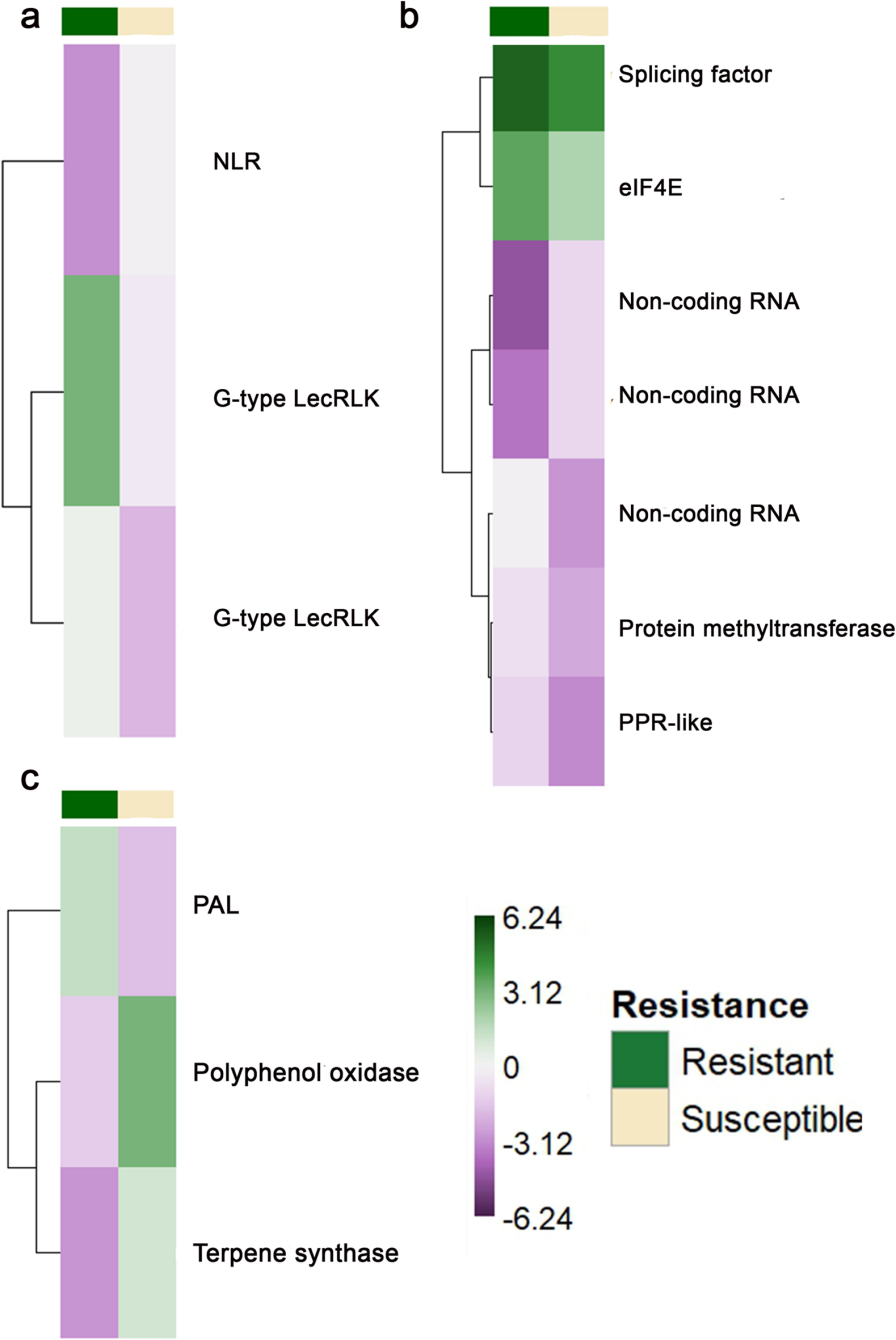
Heatmaps showing mean normalized gene expression for resistant and susceptible genotypes for stable transcriptional predictors of European canker resistance, categorized by putative gene functions: (**a**) disease resistance and defense response, (**b**) epigenetic and gene expression regulation and (**c**) secondary metabolism.

The functional annotation of the 26 stable predictors identified through the variable importance spectrum indicated that they could be classified into eight main functional categories based on putative gene function (Fig. 3; Table S3). Three genes were associated with disease resistance and defense response, including one NLR and two G-type LecRLKs. Three genes were linked to secondary metabolism, comprising one phenylalanine ammonia-lyase (PAL), one terpene synthase, and one polyphenol oxidase. Two genes were associated with cell-wall modification, including TUNICAMYCIN INDUCED 1-like and 2-hydroxy-palmitic acid dioxygenase MPO1-like. Seven transcripts were related to epigenetic and gene expression regulation, including a splicing factor (YJU2), one pentatricopeptide repeat (PPR-like) protein, one eukaryotic translation initiation factor 4E (eIF4E), one RNA methyltransferase, and three non-coding RNA transcripts. Two genes encoded transcriptional regulators, represented by AGAMOUS-like 24 and a MYB-like transcription factor. Three genes were linked to transport and membrane functions, including an ABC-2 type transporter, a mechanosensitive ion channel, and a translocase of the outer membrane (TOM22-V). Three additional genes were associated with signal transduction and kinase activity, comprising S-methyl-5-thioribose kinase, sphingosine kinase 1, and a protein kinase superfamily member. The remaining transcripts included one serine carboxypeptidase-like 20 and two genes of unknown function.

### KEGG pathway enrichment in differentially expressed genes

29% of predicted genes were annotated with KEGG pathways. The most enriched pathway in both bulk-R and bulk-S was phenylpropanoid biosynthesis (ko00940, table S4), with 146 DEGs in bulk-R and 167 in bulk-S, including genes like 4CL, peroxidases, and cinnamyl alcohol dehydrogenase (CAD). It also included genes specific to the lignin pathway such as Cinnamoyl-CoA reductase (CCR). Over 63% of these genes were upregulated in response to infection. Additional enriched pathways included biosynthesis of secondary metabolites, such as flavonoids and terpenoids, and nitrogen metabolism (ko00910). Key pathways related to environmental information processing, including ABC transporters, plant hormone signaling, and MAPK signaling, were also enriched. The cutin, suberin, and wax biosynthesis pathway (ko00073) showed enrichment, with 18 DEGs in bulk-R and 20 in bulk-S (table S4).

### KEGG pathways unique to bulks

Among within-group comparisons, the only uniquely enriched pathway in bulk-R was carbon fixation (ko00710, table S4). Bulk-S showed five unique pathways, including ascorbate metabolism (ko00053) and amino acid metabolism (ko00270, ko00400). A comparison of infected bulk-R versus bulk-S revealed enriched pathways related to β-alanine biosynthesis (ko00410) and amino acid degradation (ko00280).

### PFAM domain enrichment in differentially expressed genes

PFAM analysis annotated 81% of the predicted genes. The most significant domain in both bulks was the leucine-rich repeat N-terminal domain (LRRNT_2), followed by cytochrome p450s and UDP-glycosyltransferases (table S5). The majority of LRRNT_2 genes were putative Leucine-rich repeat RLKs. Domains such as B_lectin, S_locus_glycop, PAN_2, and DUF3403 were enriched in both bulks, with over 79% of associated DEGs upregulated. The DEGs associated with these domains are primarily G-type lectin receptor-like kinases (Sun et al. 2020). Additionally, the galacturonan-binding domain (GUB_WAK_bind) was enriched, with over 88% of related WAK/WAKL genes upregulated upon infection. DEGs with a Malectin domain were also enriched in both bulks (table S5).

### PFAM domains unique to bulks

In bulk-R, 12 unique domains were enriched, including KIP1, methyltransferases, and dynamin protein family domains (table S5). While the enrichment of these protein domains was not significant in bulk-S, the vast majority (> 85%) of these genes were also DE in bulk-S. Bulk-S had 38 unique enriched domains, including sugar efflux transporters (MtN3_slv) and the ‘Dirigent’ domain, which is involved in lignin synthesis.

### Enrichment of PFAM domains in DEGs from partially resistant vs. susceptible Apple genotypes

There were 13 significantly enriched protein domains among the 68 DEGs that could be annotated from the contrast of bulk-R infected vs. bulk-S infected (Table S5). The Toll Interleukin Receptor (TIR) domain was enriched due to five putative TIR-NBS-LRR NLRs, with four downregulated and one upregulated in bulk-R. The leucine-rich repeat domain (LRR_3) was linked to three of these NLRs. Coatomer-related domains (COPI_C, Coatomer_WDAD) were enriched, with three genes more highly expressed in bulk-S. Four PFAM domains associated with G-type lectin receptor-like kinases (B_lectin, S_locus_glycop, PAN_2, DUF3403) were also enriched, with corresponding genes located on chromosome 5.

### Transcriptome comparisons based on presence/absence of QDR-haplotype

A comparative analysis was conducted to identify candidate resistance genes for loci with a small effect on the disease resistance phenotype. The analysis was based on the presence/absence of QDR SNP-haplotypes across six QTL regions^5^ (Table 3, table S6). ‘GD’, a parent with at least one QDR haplotype per locus, was used for expression validation. Transcriptomes of QTL+ and QTL− plants were compared under infection and control conditions to assess constitutive expression differences. No significant correlations (*p* < 0.05) were found between haplotype presence at different QTL (chi-square tests).

Differential expression (DE﻿) results are summarized in Table S7, with full gene lists provided in Tables S8–S9. Key DEGs of interest are presented in Table 4, including those validated in ‘GD’, functionally similar genes within the same QTL, and genes encoding NLRs or RLKs. The complete validation dataset from ‘GD’ is available in Table S10.

**Table 3:** QTL configuration for six loci associated with European canker resistance in 25 Apple full-sibling progeny, their parents (‘Gala’ and ‘EM-Selection-4’), and ‘Golden Delicious.

Chr	QTL ID	SNP haplotype denoted QTL+	No. of QTL + individuals	No. of QTL- individuals	QTL + haplotypes in ‘Golden Delicious’
**2**	RND-QT-2	CGAAAAGAGGAGGACGGGAAGAGAAACCACAGGAGCCGCAAAAAAAGAGGGAACGAAG	18	9	1
**6**	RND-QTL6	GGAAACAGA	23	4	2
**8**	RND-QTL8	AGCGGGGCAAGAAAAGGAGGAGAGAGGGAAAAAGGGAGAGGGGAAGAAGCAAGGAGAAGGG and CAAAGGGCAGAGAGGAGGGAGAGGGAAGAAAAAGGGAGAGGGGGAAGAAAACAGGGAGGAA	22	5	1
**10**	RND-QTL10	AAGAGCAGCCGCGGGACAACAGGAGAAACGCAGAG	23	4	2
**15**	RND-QTL15	AAAAG	18	9	1
**16**	RND-QTL16	GAGGACCGAGAAACGAGGAGAGGAAGAACAAGAAAAGAAAAA	10	17	1

**Table 4:** DEGs of interest from QTL+ vs. QTL- comparison. This table displays differentially expressed genes (DEGs) that meet the following criteria: validated genes, genes with the same predicted function as a validated gene at the same QTL, or genes predicted to function as NLR or RLK. A negative log2 fold-change (LFC) indicates higher expression in QTL+ genotypes, while a positive LogFC indicates higher expression in QTL- genotypes.

Gene ID GDDH13_v1.1	Resistance QTL	LFC Control	LFC Infected	Putative gene function	DE in ‘GD’
MD02G1188900	RND-QTL2	*N.S*	−1.8	ACT domain	Up
MD02G1164500	RND-QTL2	−3.3	−2.6	Disease resistance protein (CC NBS LRR class)	-
MD02G1217100	RND-QTL2	*N.S*	1.5	Disease resistance protein (TIR NBS LRR class)	-
MD02G1260200	RND-QTL2	1.5	1.5	Disease resistance protein (TIR NBS LRR class)	-
MD02G1282000	RND-QTL2	*N.S*	−1.4	Protein kinase	-
MD02G1164900	RND-QTL2	*N.S*	−2.7	Unknown function	Up
MD02G1245800	RND-QTL2	1.0	1.2	Wall associated kinase like (WAKL)	-
MD02G1246300	RND-QTL2	1.0	1.4	Wall associated kinase like (WAKL)	-
MD02G1247400	RND-QTL2	*N.S*	−1.9	Wall associated kinase like (WAKL)	-
MD02G1234300	RND-QTL2	5.4	4.9	Wall associated kinase like (WAKL)	-
MD02G1234800	RND-QTL2	3.2	2.6	Wall associated kinase like (WAKL)	-
MD02G1246100	RND-QTL2	1.9	2.2	Wall associated kinase like (WAKL)	-
MD02G1246600	RND-QTL2	4.0	2.4	Wall associated kinase like (WAKL)	-
MD02G1274600	RND-QTL2	*N.S*	−2.0	Wall associated kinase like (WAKL)	-
MD02G1246700	RND-QTL2	2.8	2.2	Wall associated kinase like (WAKL)	-
MD02G1249500	RND-QTL2	*N.S*	−1.2	Wall associated kinase like (WAKL)	-
MD02G1273500	RND-QTL2	*N.S*	−1.3	Wall associated kinase like (WAKL)	Up
MD02G1273700	RND-QTL2	*N.S*	−1.8	Wall associated kinase like (WAKL)	Up
MD02G1249700	RND-QTL2	*N.S*	−1.4	Wall associated kinase like (WAKL)	-
MD02G1254300	RND-QTL2	*N.S*	−2.0	Wall associated kinase like (WAKL)	Down
MD02G1267000	RND-QTL2	−4.0	*N.S*	Zinc induced facilitator	Up
MD06G1099100	RND-QTL6	*N.S*	−1.1	ABC transporter	-
MD06G1069800	RND-QTL6	*N.S*	2.3	RNA-polymerase	Down
MD06G1103300	RND-QTL6	*N.S*	−3.8	UDP-Glycosyltransferase	Up
MD08G1042700	RND-QTL8	*N.S*	−2.5	Disease resistance protein (NB-ARC domain)	-
MD08G1019600	RND-QTL8	*N.S*	−1.2	Disease resistance protein (TIR-NBS-LRR class)	-
MD08G1020000	RND-QTL8	*N.S*	−1.8	Disease resistance protein (TIR-NBS-LRR class)	-
MD08G1055100	RND-QTL8	*N.S*	−1.2	Glutathione peroxidase	Up
MD08G1064100	RND-QTL8	*N.S*	1.5	Heat shock factor protein	Down
MD08G1026800	RND-QTL8	*N.S*	1.6	Heavy metal associated isoprenylated plant protein (HIPP)	Down
MD10G1238200	RND-QTL10	*N.S*	−2.5	NAC transcription factor	Up
MD10G1250000	RND-QTL10	−5.6	−3.4	Wall associated kinase (WAK)	-
MD10G1250500	RND-QTL10	−2.9	*N.S*	Wall associated kinase (WAK)	-
MD10G1251200	RND-QTL10	−4.9	−2.2	Wall associated kinase (WAK)	-
MD15G1102100	RND-QTL15	−1.3	*N.S*	ABC transporter	Up
MD15G1090400	RND-QTL15	2.8	3.6	Disease resistance protein (CC NBS LRR class)	-
MD15G1090100	RND-QTL15	2.7	2.9	Disease resistance protein (LRR and NB-ARC domains)	-
MD15G1090000	RND-QTL15	3.2	3.5	Disease resistance protein (NB-ARC domain)	-
MD15G1090300	RND-QTL15	3.5	4.2	Disease resistance protein (NB-ARC domain)	-
MD15G1179700	RND-QTL15	*N.S*	−2.0	Disease resistance protein (TIR NBS LRR class)	-
MD15G1073400	RND-QTL15	*N.S*	2.3	Epimerase	-
MD15G1073500	RND-QTL15	4.1	3.5	Epimerase	Down
MD15G1103500	RND-QTL15	−3.1	−2.1	LisH-domain	Down
MD15G1061900	RND-QTL15	−3.0	−3.5	PITH domain-containing protein	Up
MD15G1077600	RND-QTL15	1.4	*N.S*	Transcription factor	Down
MD15G1103400	RND-QTL15	−1.6	−1.4	Transcriptional co-repressor/LisH domain	-
MD16G1112900	RND-QTL16	*N.S*	−2.1	AMP dependent synthetase and ligase	Up
MD16G1113000	RND-QTL16	−2.3	−2.4	AMP-dependent synthetase and ligase	-
MD16G1104200	RND-QTL16	2.0	*N.S*	Cytochrome P450	-
MD16G1104300	RND-QTL16	2.0	*N.S*	Cytochrome P450	-
MD16G1116300	RND-QTL16	−3.2	*N.S*	Cytochrome P450	Up
MD16G1055500	RND-QTL16	*N.S*	2.6	D-aminoacyl tRNA deacylases	Up
MD16G1082200	RND-QTL16	*N.S*	3.0	Disease resistance protein (TIR NBS LRR class)	-
MD16G1125800	RND-QTL16	*N.S*	−1.2	NAC transcription factor	Up
MD16G1072500	RND-QTL16	−4.1	−4.0	Transmembrane amino-acid transporter	Down

### QTL interaction network: genes associated with multiple QDR-haplotypes

**Fig. 4:**
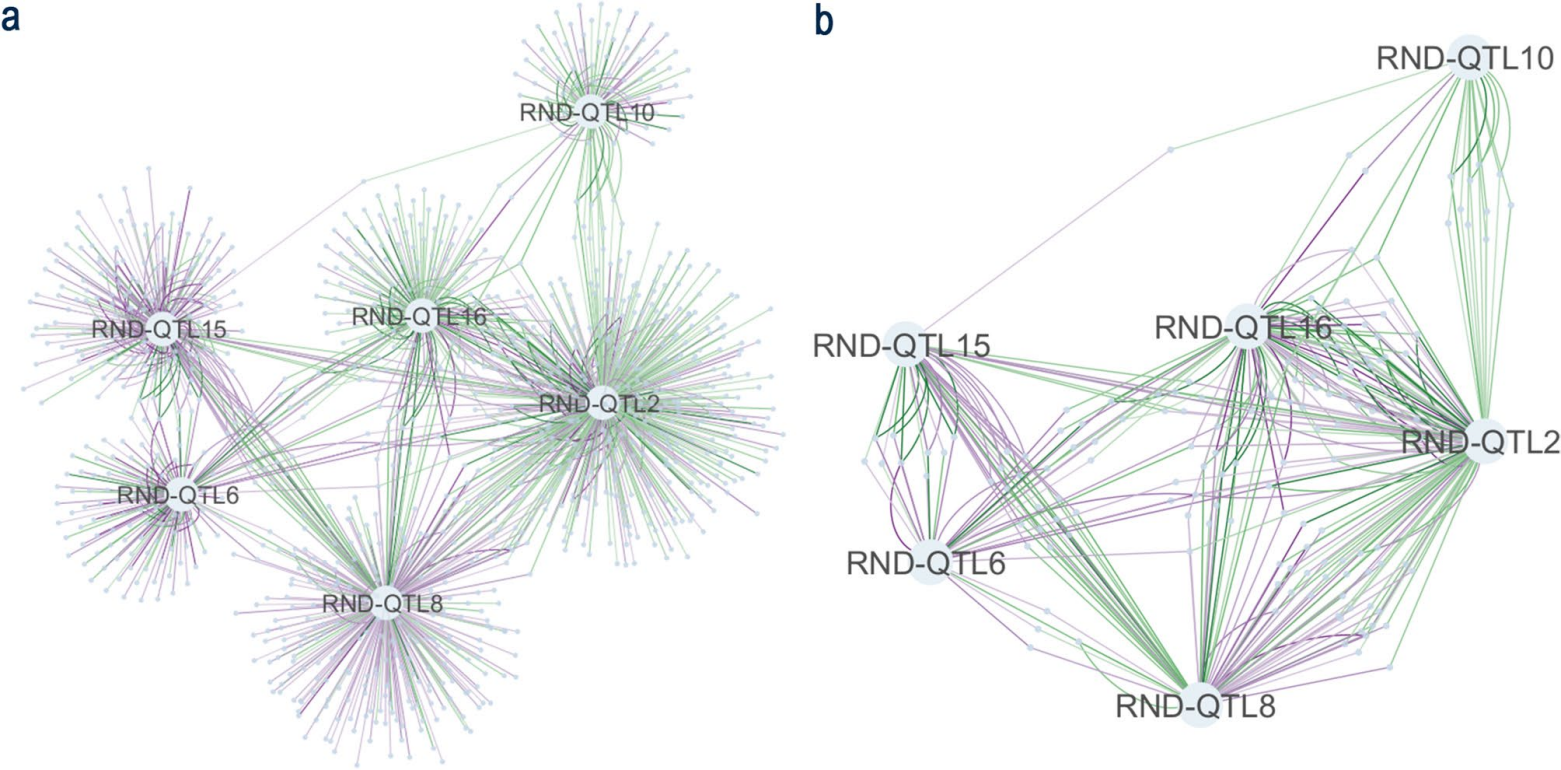
QTL interaction network showing differentially expressed transcripts from a comparative analysis of gene expression in infected trees based on presence/absence of SNP-haplotypes associated with quantitative disease resistance to *N. ditissima.* Purple indicates higher expression in QTL+ and green indicates higher expression QTL-. **A**) All DEGs from the analysis of six QTL, **B**) Subset of DEGs identified in more than one comparative analysis of QTL-haplotype.

To investigate interactions between QTLs, we investigated genes that were DE in multiple QTL+/QTL- contrasts for infected trees. A total of 147 DEGs were associated with more than one QDR haplotype (Fig. 4, Table S11), indicating potential cross-talk between resistance loci.

Genes involved in the phenylpropanoid pathway were differentially expressed across multiple QTLs, including 4CL (*MD16G1112900*,* MD16G1113000*) on chr 16 and PAL (*MD03G1121400*,* MD03G1121500*) on chr 3. These genes showed varied expression patterns across resistance backgrounds.

Pathogen recognition and signaling genes were also affected, with seven NLRs (chr 2, 3, 10, 15, 16) and two WAKLs (chr 2) showing differential expression in plants carrying resistance alleles.

Ten transcripts, including a Zinc-finger protein (*MD11G1125800)*, eIF4E (*MD10G1268700*), and a Ca²⁺-binding EF-hand protein (*MD10G1306200*), were downregulated in both RND-QTL10 + and RND-QTL2 + genotypes.

Fifteen genes exhibited opposing expression patterns between RND-QTL8 + and RND-QTL15+, including genes involved in mRNA splicing (*MD15G1111800*,* MD15G1111000*,* MD15G1111900*), chromatin modification (*MD15G1111800*,* MD15G1076200*), and pathogen response (NLR *MD03G1202400;* LRR-RLK *MD03G1072600*).

Additionally, 33 transcripts showed inverse expression patterns between RND-QTL8 + and RND-QTL2+, including genes involved in disease resistance (*MD08G1042700*,* MD02G1042000)*, jasmonate signaling (*MD08G1027300*), H₂O₂ production (*MD13G1087700*), calcium signaling (*MD03G1005600*), and ribosomal function (*MD02G1015900*,* MD02G1001700*).

## Discussion

This study utilized transcriptomes from 25 full-sibling apple progeny and their two parents, ‘Gala’ and EM-Selection-4, to investigate gene expression during infection by the canker pathogen *N. ditissima.* The progeny, part of a multi-parental population aimed at identifying canker resistance QTL, segregated for six out of seven QTL associated with partial resistance to this pathogen^5^, Through RNA-Seq, key DEGs and pathways contributing to resistance mechanisms were identified. The results from Random Forest classification, functional annotation, KEGG pathway enrichment, and PFAM domain analysis have highlighted candidate genes and pathways linked to host defence responses.

DE and functional enrichment analyses revealed that genes in the phenylpropanoid pathway were differentially regulated during canker infection in both bulk-R and bulk-S, with over 63% showing increased abundance post-infection. Protein domains related to peroxidases and laccases (Cu-oxidases) were enriched, which is notable as both enzymes contribute to lignin polymerization^38^. Two PAL genes on chromosome 3, *MD03G1121500* and *MD03G1121400*, were identified from the analysis. The former was one of the stable predictors of resistance, while the latter was DE in comparisons of RND-QTL16+/-. PAL, a key enzyme in the phenylpropanoid pathway, converts phenylalanine into trans-cinnamic acid, a precursor for lignin and flavonoid biosynthesis. Both PAL genes showed higher expression in bulk-R, indicating increased activity in the partially resistant apple genotypes, particularly in those with RND-QTL8 + and RND-QTL16 + haplotypes, suggesting regulation downstream of genetic variations conferring resistance.

Further evidence for the role of the phenylpropanoid pathway in partial resistance to *N. ditissima* includes two putative 4CL genes (*MD16G1112900* and *MD16G1113000*) located within the QTL interval on chromosome 16. Both genes were significantly more expressed in apple progeny with the RND-QTL16 + haplotype, and one also DE in GD. 4CL is a key enzyme that catalyzes the conversion of hydroxycinnamates into CoA esters for lignin and flavonoid biosynthesis^39^, and its activity has been linked to pathogen resistance in multiple crops^40–42^. Additionally, three putative CYP genes were identified within RND-QTL16, involved in secondary metabolite synthesis^43^. These findings suggest a shift in phenylpropanoid gene expression and altered lignin accumulation via peroxidase and laccase activity in response to *N. ditissima* infection. However, further studies are needed to assess the relative contributions of lignin biosynthesis and phenylpropanoids to quantitative disease resistance to European canker. Higher lignin levels generally correlate with increased resistance, especially against vascular pathogens such as *Fusarium*,* Xanthomonas*,* and Verticillium* that spread through the xylem^44^.

*N. ditissima* infection triggered differential expression of genes involved in pathogen recognition, including pathogenesis-related proteins, NLRs, and RLKs. Our findings suggest that apple recognises *N. ditissima* through a combination of basal immunity and specialised NLRs. However, it remains unclear whether NLRs contribute to QDR or if the pathogen exploits them as susceptibility factors^8^. One NLR on chromosome 10, *MD10G1137400*, were among the top predictors of canker resistance. *MD10G1137400* showed significant expression differences between bulk-R and bulk-S, regardless of infection and exhibited lower expression in resistant progeny, suggesting that downregulation may be a defense strategy to prevent pathogen exploitation of the immune response.

G-type LecRLKs emerged as potentially having an important role in the apple defense to European canker. G-type LecRLKs play key roles in plant immunity, growth, and development^45^. As a subgroup of RLKs with a lectin domain for carbohydrate recognition, they are crucial in cell signaling, but also in plant defense^45^. Two of these genes, both located on chromosome 5, showed higher expression in bulk-R genotypes following infection and were among those with stable predictive ability for canker resistance. G-type LecRLKs have been shown to positively regulate chitin signalling in the interaction between *Nicotiana benthamiana* and *Sclerotinia sclerotiorum*^46^ as well as regulating immunity activated by the recognition of nlp20 (Necrosis and ethylene-inducing peptide 1-like proteins), a group of proteins derived from certain pathogen and especially important in the pathogenesis of necrotrophic pathogens^47^.

In addition to above mentioned groups of genes there was also an enrichment of protein domains like malectin, COPI_C, Coatomer_WDAD, and ECH_2 in differentially expressed genes from bulk-R vs. bulk-S infected trees, suggesting roles in intracellular signaling and metabolic adaptation during pathogen attack. Malectin supports stress responses and protein quality control^48^, while COPI_C and Coatomer_WDAD are involved in vesicle trafficking for defense molecule secretion^49^. Additionally, ECH_2 enzymes are involved in auxin metabolism, and their role in peroxisomal fatty acid β-oxidation affects the generation of jasmonic acid precursors and can contributes to reactive oxygen species (ROS) production^50^.

Seven out of the 26 genes most predictive of canker resistance were genes or transcripts with a putative role in epigenetic regulation and gene expression, including roles in RNA splicing, translation initiation, and non-coding RNA regulation. Interestingly several putative ncRNA were found in this group. An increasing number of ncRNAs have been identified as key players in plant immunity, though the mechanistic details are still limited^51^. These ncRNAs regulate various aspects of immunity, including pathogen perception, signal transduction, and immune responses, through strategies such as modulating gene expression, interacting with proteins, and working in concert with other ncRNAs. A single ncRNA can target multiple genes, potentially influencing not only immunity but also plant development and responses to abiotic stresses.

We identified several candidate genes involved in pathogen interactions within QTLs associated with partial resistance to *N. ditissima* in apple^5^. Clusters of putative WAKs and WAKLs were found on chromosomes 10 and 2, respectively. WAKs typically contain serine/threonine kinase, epidermal growth factor (EGF), and galacturonan-binding (GUB) domains, while WAKLs generally lack the EGF domain^9^. These RLKs regulate plant growth and stress responses, often enhancing immunity but occasionally suppressing resistance^9,52,53^. In apple, WAKs show both positive and negative regulation in response to pathogens^54^. Among candidate genes in QTL10, three WAKs had lower expression in QDR-allele trees, with two showing upregulation under both control and infected conditions. A cluster of 14 WAKLs in QTL2 included two (*MD02G1273500* and *MD02G1273700*) with higher expression in QTL2-R trees and significant upregulation in ‘GD’ upon infection.

A putative HIPP gene within the QTL on chr 8 showed lower expression in QTL-R trees and was downregulated in ‘GD’. HIPPs are susceptibility targets of necrotrophic pathogens^55^ and nematodes^56^. *Oryza sativa* HIPP05 (*Pi21*) is a well-known susceptibility factor for *Magnaporthe oryzae*, where loss-of-function mutations confer resistance, while overexpression in Arabidopsis increases pathogenicity^57,58^.

The transcript of a putative ncRNA, MD10G1176800, was linked to both QTLs on chromosomes 2 and 10, with higher abundance in individuals carrying the QTL + genotype. It was located on chromosome 10, 57 kbp from the predicted QTL region.

This study used a transcriptome approach to identify candidate genes linked to multiple resistance QTL for European canker in apple. However, only a subset of DE genes between QTL-R and QTL-S plants could be validated in ‘GD’, despite its QDR alleles. This may be due to differences in infection stage at sampling, which significantly influences gene expression, or the validation focusing only on infection-induced genes, excluding constitutively expressed ones.

Some resistance QTL genes may have gone undetected due to the DE analysis focusing on single alleles and ignoring background QTL effects, especially if the QTL has a minor impact on disease progression. Differences in haplotype alleles for QTL 8 suggest potential genetic variation in resistance, and the low representation of genotypes lacking QDR alleles for QTL6 and QTL8 may have reduced detection power. Despite these limitations, this study identified candidate genes that, with further functional validation, could aid in breeding canker-resistant apple varieties.

Our results suggest that the partial resistance QTLs may act additively at the transcriptional level. Several genes, including *PAL*, *4CL*, and multiple immune receptors, were differentially expressed in association with more than one QDR haplotype. In total, 147 transcripts showed expression differences linked to multiple QTLs, indicating shared regulatory effects across loci.

While we did not model expression relative to allele dosage, the overlap in DEGs across QTL contrasts, and the increased expression of defense-related genes in individuals carrying multiple QTL + haplotypes, supports additive modulation of resistance pathways. However, we also observed opposing expression patterns between certain QTLs, suggesting potential complexity in their interactions. Overall, these findings point to cumulative, and in some cases interactive, effects of QTLs on defense gene expression.

We used a Random Forest variable selection approach to reduce complexity and highlight gene expression patterns that differ between resistant and susceptibe responses to *N. ditissima* infection in apple. ML techniques help address challenges such as handling large datasets, recognizing patterns, and optimizing models, improving the efficiency and accessibility of analyzing complex biological systems^59^. In human transcriptomics ML is commonly used for RNA-seq data to predict disease states, identify key transcripts, discover disease biomarkers, determine differentially expressed genes and deconvolve single-cell data^59^. However, there are only limited examples of ML being used as a tool to understand and predict host-pathogen interactions in plants. Sia et al.^19^ used ML to predict Arabidopsis transcriptomic responses to multiple pathogens and identified key gene sets predictive of disease development through feature selection. Similarily to our study Panahi et al.^20^ used a combination of RNA-Seq and ML to rank differentially expressed genes associated with *Rhizoctonia solani* resistance in sugar beet in order to identify key biomarkers of resistance.

Feature selection in RF can be unreliable for identifying candidate genes due to variability in selected features, bias toward strong predictors, and the exclusion of correlated genes, potentially missing biologically significant candidates. To address this, we applied a cross-validated stability selection approach based on repeated estimation of variable importance across genotype-blocked folds. This method identified a consistent subset of genes with stable predictive ability for canker resistance. The resulting gene set was highly predictive of disease resistance in the ‘Golden Delicious’ and ‘M9’ varieties. However, further testing in a broader range of cultivars is needed to assess whether these genes are specific to certain genetic backgrounds. Additionally, it is important to note that canker resistance is a complex trait, not a binary response, with many cultivars exhibiting moderate resistance.

This study provides valuable insights into the genetic and molecular basis of partial resistance to *N. ditissima* in apple, offering potential targets for breeding canker-resistant varieties. By integrating transcriptomic analysis with QTL mapping and machine learning-based feature selection, we identified candidate genes and pathways that contribute to host defense. Notably, the phenylpropanoid pathway, immune receptors, and epigenetic regulators emerged as key components of the resistance response.

The identification of putative resistance-associated genes within QTL regions highlights opportunities for marker-assisted selection, potentially accelerating the development of resistant cultivars. In particular, PAL and 4CL genes linked to lignin biosynthesis may serve as biomarkers for enhanced structural defense, while NLRs and RLKs could be further explored for their roles in pathogen recognition and signaling.

Despite challenges such as the complex genetic architecture of resistance and variability in gene expression, this study underscores the power of transcriptomics and machine learning in dissecting quantitative disease resistance. Future work should focus on functional validation of candidate genes and their integration into breeding programs, ensuring their effectiveness across diverse genetic backgrounds. By refining selection strategies, these findings can contribute to the development of apple varieties with improved resilience against European canker, ultimately supporting sustainable apple production.

## Supplementary Information

Below is the link to the electronic supplementary material.

Supplementary Material 1

## Data Availability

The raw sequence data underlying this article is available in NCBI (BioProject accession number: PRJNA1055417) at [https://www.ncbi.nlm.nih.gov/sra/PRJNA1055417].
